# The Predictive Value of Serum NT-proBNP on One-Year All-Cause Mortality in Geriatrics Hip Fracture: A Cohort Study

**DOI:** 10.7759/cureus.45398

**Published:** 2023-09-17

**Authors:** Bin-Fei Zhang, Shang-Bo Ren, Ming-Xu Wang

**Affiliations:** 1 School of Public Health, Xi'an Jiaotong University, Xi'an, CHN; 2 Department of Joint Surgery, Honghui Hospital, Xi'an Jiaotong University, Xi'an, CHN

**Keywords:** nt-probnp, regression, risk factor, mortality, hip fracture

## Abstract

Objective

This study evaluated the association between N-terminal prohormone of brain natriuretic peptide (NT-proBNP) concentration and one-year mortality in geriatric patients with intertrochanteric and femoral neck fractures receiving the operative treatment.

Methods

Consecutive age ≥65 years patients with hip fractures were screened between January 2015 and September 2019. Demographic and clinical characteristics of the patients were collected. The multivariate logistic regression models were used to identify the association between preoperative NT-proBNP concentrations and mortality. All analyses were performed using EmpowerStats and the R software.

Result

One thousand two hundred nineteen patients were included in the study. The average age was 79.73±6.65 years (range 66-99 years). The mean NT-proBNP concentration was 616.09±1086.85 ng/L (median 313.40 ng/L, range 16.09-20123.00 ng/L). The follow-up was 35.39±15.09 months (median 35.78 months, range 0.10-80.14 months). One hundred and eleven (9.1%) patients died within one year. After adjusting for confounding factors, multivariate logistic regression models showed a curved association between preoperative NT-proBNP concentration and one-year mortality. When the NT-proBNP concentration was below 1099 ng/L, the mortality increased by 10% (OR=1.10, 95%CI: 1.03-1.17, *P*=0.0025) when NT-proBNP increased by 100 ng/L. When the NT-proBNP concentration was above 1099 ng/L, the mortality did not increase anymore when NT-proBNP increased (OR=1.00, 95%CI: 0.99-1.02, *P*=0. 7786). Thus, NT-proBNP was a valuable indicator to predict high one-year mortality in practice.

Conclusion

The NT-proBNP concentrations were nonlinearly associated with mortality in elderly hip fractures with a saturation effect, and NT-proBNP was a risk indicator of all-cause mortality. A well-designed controlled trial to show the role of mortality by decreasing the concentration of NT-proBNP is needed in the future.

## Introduction

Hip fractures are the most devastating type of osteoporotic fracture, associated with increased risk of morbidity, mortality, disability, and a heavy burden on social healthcare costs, especially for the geriatric population [1]. Mortality is the most severe outcome for geriatric patients with hip fractures after discharge. Previous studies have indicated that many factors are associated with short-term or long-term mortality, such as aged > 75 [2,3], male [4], comorbidities (heart failure, low ejection fraction, pulmonary, chronic kidney disease) [2-5], delay in operation [3,6], and fracture type [3].

In addition, some serum biomarkers are associated with the outcomes of hip fractures, such as lactate concentration [7], anion gap [8], and methyl-CpG binding protein 2 [9]. Cardiac myocytes secreted the N-terminal prohormone of brain natriuretic peptide (NT-proBNP) [10]. It has a longer half-life and high serum concentration, and the physiological characteristics of NT-proBNP make it more versatile and accurate in clinical applications. Determination of NT-proBNP concentration in the serum can be used to evaluate heart function. In clinical practice, patients with NT-proBNP ≥ 450 ng/L had a 2.92-fold incidence of cardiovascular disease or 3.81-fold heart failure compared to individuals with NT-proBNP < 125 ng/L of normal value [11]. It is reported that preoperative NT-proBNP > 3984 ng/L is an independent predictor of cardiac events in high-risk patients undergoing acute hip fracture surgery [12], and abnormally elevated NT-proBNP significantly increased all-cause mortality within 90 days after surgery [13]. In addition, Nordling et al. found that an elevated perioperative NT-proBNP concentration is common in hip fracture patients. In a blinded prospective cohort study, it is an independent predictor of short-term and long-term mortality superior to the common American Society of Anesthesiologists's physical status classification [14]. This indicates that NT-proBNP concentration may have an association with postoperative mortality. However, few studies have assessed the relationship between NT-proBNP and mortality in geriatric hip fractures and whether it could predict prognosis or mortality.

In this cohort study, we aimed to identify the role of NT-proBNP concentrations on mortality in hip fractures, intending to identify a clinically useful biomarker.

## Materials and methods

Study design

The Ethics Committee of Xi’an Honghui Hospital approved this study (No. 202201009, approval on January 28, 2022).

We screened the patients with surgically treated hip fractures between January 1, 2015, and September 30, 2019, from the electronic medical record system. The study was conducted according to strengthening the reporting of cohort, cross-sectional and case-control studies in surgery (STROCSS) 2021 guidelines [15]. All human-related procedures followed the Declaration of Helsinki. We contacted and followed the patients by telephone. Oral informed consent was obtained from patients or their legal guardian(s) by telephone.

Participants

The inclusion criteria were as follows: 1) age ≥65 years; 2) diagnosis of femoral neck or intertrochanteric fracture, suffered from high or low impact; 3) patients who were receiving the operative treatment; 4) patients could be contacted by telephone. Patients who could not be contacted and those with malignancy or secondary to metastatic disease were excluded from this study [16,17].

Hospital treatment

Ultrasonography was used to examine the cardiac function and screen lower extremity deep vein thrombosis. Intertrochanteric fractures received closed or open reduction and internal fixation [closed reduction internal fixation (CRIF)/ open reduction internal fixation (ORIF)] by proximal femoral intramedullary nailing. Femoral neck fractures were often treated with hemiarthroplasty (HA) or total hip arthroplasty (THA), according to the patient’s situation [16,17]. Patients with high activity level requirements and large fracture displacements usually receive THA.

Variables

The endpoint was one-year mortality, and serum NT-proBNP concentration at admission was the independent variable. The criteria for internal diseases were based on their last guidelines.

Statistical analysis

Continuous variables are reported as mean±standard deviation (SD), or median (min, max), and categorical variables are presented as frequencies and percentages. We used χ2, a one-way analysis of variance, or the Kruskal-Wallis H test to test for differences among different NT-proBNP concentrations [17]. We performed univariate and multivariable logistic regression models to test the association between NT-proBNP concentration and one-year mortality. We also use a generalized additive (penalized spline model) and smooth curve fitting to address nonlinearity. The two-piecewise binary logistic regression model was used to find the inflection point on the smooth curve. All analyses were performed using statistical software packages R (http://www.R-project.org, R Foundation) and EmpowerStats (http://www.empowerstats.com, X&Y Solutions Inc., Boston, MA, USA). Odds ratios (OR) and 95% confidence interval (CI) were calculated. Statistical significance was set at a P-value <0.05 (two-sided).

## Results

Patient characteristics

**Figure 1:**
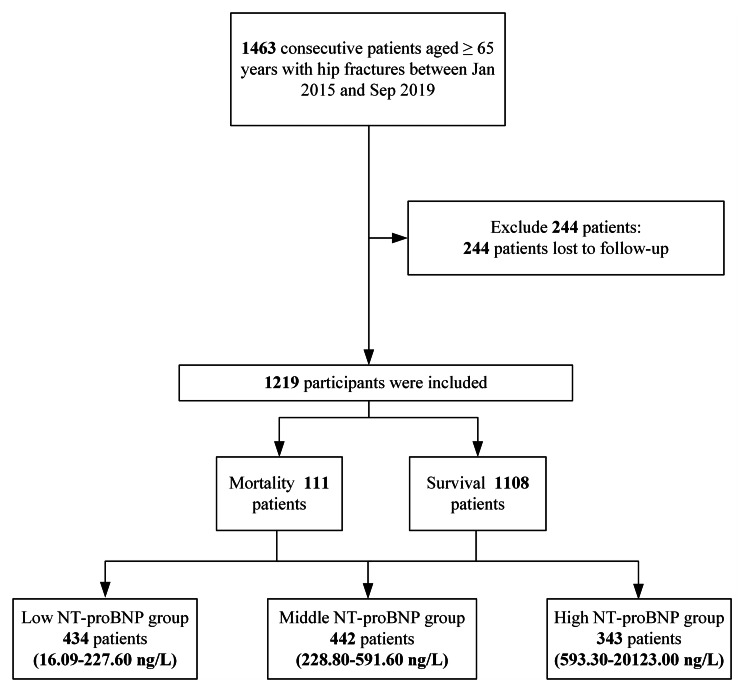
Study flow diagram of low, middle, and high NT-proBNP groups.

We screened 1463 consecutive participants surgically treated for a hip fracture in the system. Two hundred forty-four patients (16.7%) were lost to follow-up. One thousand two hundred and nineteen participants were included in this study, including 365 males and 854 females. The flow chart is shown in Figure 1. The average age was 79.73±6.65 years (range 66-99 years). The mean NT-proBNP concentration was 616.09±1086.85 ng/L (median 313.40 ng/L, range 16.09-20123.00 ng/L). The follow-up was 35.39±15.09 months (median 35.78 months, range 0.10-80.14 months). One hundred and eleven (9.1%) patients died within one year. We divided the patients into three groups according to NT-proBNP concentrations. Table 1 lists the demographic and clinical characteristics of all 1219 patients, including comorbidities, factors associated with injuries, and surgery strategies.

**Table 1:** Demographic and clinical characteristics. The data is represented as N (%); Mean±SD. P<0.05 is considered significant. P-value*: Kruskal-Wallis rank-sum test or Fisher’s exact probability test. NT-proBNP: N-terminal prohormone of brain natriuretic peptide; CRIF/ORIF: closed/open reduction and internal fixation; HA: hemiarthroplasty; THA: total hip arthroplasty; CHD: coronary heart disease; COPD: chronic obstructive pulmonary disease.

NT-proBNP tertiles		Low	Middle	High	P-value	P-value*
		16.09-227.60 ng/L	228.80-591.60 ng/L	593.30-20123.00 ng/L		
	N	434	442	343		
	Age (year)	76.69±6.23	81.04±6.42	81.90±6.05	<0.001	<0.001
	NT-proBNP (ng/L)	138.65±55.32	368.92±104.81	1538.72±1722.47	<0.001	<0.001
	Sex					
	Male	143 (32.95%)	127 (28.73%)	95 (27.70%)	0.223	-
	Female	291 (67.05%)	315 (71.27%)	248 (72.30%)		
	Injury mechanism					
	Falling	417 (96.08%)	435 (98.42%)	331 (96.50%)	0.011	0.005
	Accident	17 (3.92%)	4 (0.90%)	8 (2.33%)		
	Other	0 (0.00%)	3 (0.68%)	4 (1.17%)		
	Fracture classification					
	Intertrochanteric fracture	306 (70.51%)	301 (68.10%)	215 (62.68%)	0.065	-
	Femoral neck fracture	128 (29.49%)	141 (31.90%)	128 (37.32%)		
	Hypertension	222 (51.15%)	220 (49.77%)	196 (57.14%)	0.101	-
	Diabetes	99 (22.81%)	72 (16.29%)	69 (20.12%)	0.051	-
	Arrhythmia	120 (27.65%)	158 (35.75%)	146 (42.57%)	<0.001	-
	Hemorrhagic stroke	9 (2.07%)	15 (3.39%)	5 (1.46%)	0.184	-
	CHD	217 (50.00%)	256 (57.92%)	213 (62.10%)	0.002	-
	Dementia	14 (3.23%)	29 (6.56%)	18 (5.25%)	0.075	-
	Ischemic stroke	137 (31.57%)	141 (31.90%)	104 (30.32%)	0.887	-
	Cancer	11 (2.53%)	10 (2.26%)	12 (3.50%)	0.550	-
	Multiple injuries	31 (7.14%)	41 (9.28%)	24 (7.00%)	0.390	-
	COPD	21 (4.84%)	30 (6.79%)	27 (7.87%)	0.210	-
	Hepatitis	13 (3.00%)	17 (3.85%)	10 (2.92%)	0.704	-
	Gastritis	7 (1.61%)	10 (2.26%)	4 (1.17%)	0.492	-
	Urea (mmol/L)	6.08±2.10	6.65±2.68	7.44±3.60	<0.001	<0.001
	Creatinine (μmol/L)	63.05±17.48	68.26±23.84	77.32±37.15	<0.001	<0.001
	Treatment Strategy					
	CRIF/ORIF	310 (71.43%)	303 (68.55%)	215 (62.68%)	<0.001	-
	HA	110 (25.35%)	138 (31.22%)	126 (36.73%)		
	THA	14 (3.23%)	1 (0.23%)	2 (0.58%)		
	Operation time (mins)	101.11±37.97	95.85±37.69	95.03±33.02	0.036	0.011
	Blood loss (mL)	233.96±134.84	238.14±136.67	241.91±141.23	0.733	0.508
	Infusion (mL)	1686.69±412.93	1583.69±381.11	1551.61±380.06	<0.001	<0.001
	Stay in hospital (d)	8.34±2.78	8.72±3.20	9.57±3.77	<0.001	<0.001
	Follow up (m)	37.64±14.19	36.02±15.09	31.73±15.56	<0.001	<0.001
	One-year mortality					
	Survival	409 (94.24%)	407 (92.08%)	292 (85.13%)	<0.001	-
	Death	25 (5.76%)	35 (7.92%)	51 (14.87%)		

Univariate analysis

The univariate analysis was used to identify potential confounding factors related to mortality (Table 2). The following variables were considered in the multivariate logistic regression: age, sex, hypertension, coronary heart disease (CHD), cancer, hepatitis, urea, creatinine, transfusion, and stay in hospital, according to the criteria of P<0.1.

**Table 2:** Effects of factors on mortality measured by univariate analysis. The data is represented as N (%); Mean±SD. P<0.05 is considered significant. NT-proBNP, N-terminal prohormone of brain natriuretic peptide; CRIF/ORIF: closed/open reduction and internal fixation; HA: hemiarthroplasty; THA: total hip arthroplasty; CHD: coronary heart disease; COPD: chronic obstructive pulmonary disease. *Note: NT-proBNP values were analyzed per each 100 ng/L increase.

One-year mortality	Survival	Death	Total	OR (95%)	P-value
Age (year)	79.48±6.63	82.28±6.37	79.73±6.65	1.07 (1.04, 1.10)	<0.0001
Sex					
Male	322 (29.06%)	43 (38.74%)	365 (29.94%)	1.0	
Female	786 (70.94%)	68 (61.26%)	854 (70.06%)	0.65 (0.43, 0.97)	0.0349
NT-proBNP (ng/L)	577.78±1028.72	998.54±1505.76	616.09±1086.85	1.02 (1.01, 1.03)*	0.0019
Injury mechanism					
Falling	1077 (97.20%)	106 (95.50%)	1183 (97.05%)	1.0	
Accident	27 (2.44%)	2 (1.80%)	29 (2.38%)	0.75 (0.18, 3.21)	0.7009
Other	4 (0.36%)	3 (2.70%)	7 (0.57%)	7.62 (1.68, 34.50)	0.0084
Fracture classification					
Intertrochanteric fracture	743 (67.06%)	79 (71.17%)	822 (67.43%)	1.0	
Femoral neck fracture	365 (32.94%)	32 (28.83%)	397 (32.57%)	0.82 (0.54, 1.27)	0.3785
Hypertension	571 (51.53%)	67 (60.36%)	638 (52.34%)	1.43 (0.96, 2.13)	0.0771
Diabetes	214 (19.31%)	26 (23.42%)	240 (19.69%)	1.28 (0.80, 2.03)	0.3003
CHD	613 (55.32%)	73 (65.77%)	686 (56.28%)	1.55 (1.03, 2.34)	0.0356
Arrhythmia	384 (34.66%)	40 (36.04%)	424 (34.78%)	1.06 (0.71, 1.60)	0.7712
Hemorrhagic stroke	384 (34.66%)	40 (36.04%)	29 (2.38%)	1.16 (0.34, 3.88)	0.8146
Ischemic stroke	347 (31.32%)	35 (31.53%)	382 (31.34%)	1.01 (0.66, 1.54)	0.9631
Cancer	27 (2.44%)	6 (5.41%)	33 (2.71%)	2.29 (0.92, 5.67)	0.0737
Multiple injuries	88 (7.94%)	8 (7.21%)	96 (7.88%)	0.90 (0.42, 1.91)	0.7841
Dementia	52 (4.69%)	9 (8.11%)	61 (5.00%)	1.79 (0.86, 3.74)	0.1205
COPD	70 (6.32%)	8 (7.21%)	78 (6.40%)	1.15 (0.54, 2.46)	0.7153
Hepatitis	33 (2.98%)	7 (6.31%)	40 (3.28%)	2.19 (0.95, 5.08)	0.0670
Gastritis	19 (1.71%)	2 (1.80%)	21 (1.72%)	1.05 (0.24, 4.58)	0.9465
Urea (mmol/L)	6.56±2.67	7.69±4.03	6.66±2.84	1.11 (1.05, 1.18)	0.0001
Creatinine (μmol/L)	68.28±26.46	74.88±31.35	68.88±27.00	1.01 (1.00, 1.01)	0.0221
Treatment Strategy					
CRIF/ORIF	749 (67.60%)	79 (71.17%)	828 (67.92%)	1.0	
HA	343 (30.96%)	31 (27.93%)	374 (30.68%)	0.86 (0.55, 1.32)	0.4861
THA	16 (1.44%)	1 (0.90%)	17 (1.39%)	0.59 (0.08, 4.53)	0.6140
Operation time (mins)	97.27±36.75	99.68±35.30	97.49±36.61	1.00 (1.00, 1.01)	0.5106
Blood loss (mL)	237.72±139.64	237.78±111.75	237.73±137.25	1.00 (1.00, 1.00)	0.9967
Transfusion (U)	1.00±1.22	1.21±1.18	1.02±1.22	1.15 (0.98, 1.33)	0.0824
Infusion (mL)	1616.50±397.17	1564.35±387.00	1611.67±396.37	1.00 (1.00, 1.00)	0.1927
Stay in hospital (d)	8.76±3.22	9.48±3.70	8.82±3.27	1.06 (1.01, 1.12)	0.0284

Multivariable analysis 

When NT-proBNP concentration was a continuous variable, the fully adjusted model showed that NT-proBNP was not associated with mortality [odds ratio (OR)=1.01, 95%CI: 1.00-1.03, P=0.0504]. When NT-proBNP concentration was divided into three groups, as shown in Table 3, we found the mortality was higher in the high concentration than the low concentration (OR=1.97; 95%CI: 1.14, P=0.0150). In addition, the P for trend also showed a linear correlation in the three models (P<0.05). However, we found that the overall result and the changing interval varied among the subgroups of NT-proBNP was unstable, and the nonlinear correlation should be explored and identified.

**Table 3:** Univariate and multivariable results by linear regression. Outcome variable: one-year mortality, Exposure variable: NT-proBNP; NT-proBNP tertiles; NT-proBNP tertiles, Non-adjusted model adjust for: none, Adjust Ⅰ model adjust for: age, sex, Adjust Ⅱ model adjust for: age, sex, hypertension, CHD, cancer, hepatitis, urea, creatinine, transfusion and stay in hospital. *Note: NT-proBNP values were analyzed per each 100 ng/L increase. P<0.05 is considered significant.

Exposure	Non-adjusted		Adjust Ⅰ		Adjust Ⅱ	
	OR (95%CI)	P-value	OR (95%CI)	P-value	OR (95%CI)	P-value
NT-proBNP	1.02 (1.01, 1.03)	0.0019*	1.02 (1.01, 1.03)	0.0055*	1.01 (1.00, 1.03)	0.0504*
NT-proBNP tertiles						
Low	1.0		1.0		1.0	
Middle	1.41 (0.83, 2.39)	0.2079	1.14 (0.66, 1.99)	0.6314	1.10 (0.63, 1.92)	0.7350
High	2.86 (1.73, 4.72)	<0.0001	2.28 (1.35, 3.87)	0.0022	1.97 (1.14, 3.41)	0.0150
P for trend		<0.0001		0.0010		0.0094

Curve fitting and analysis of the nonlinear association

**Figure 2:**
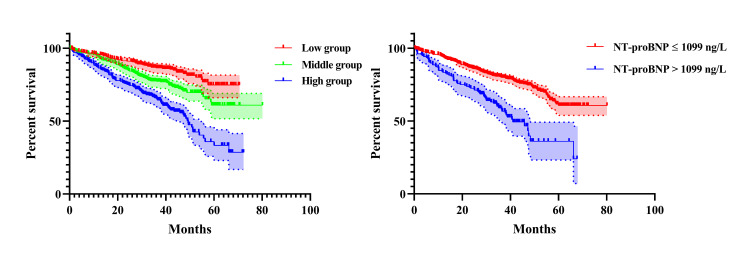
The Kaplan-Meier survival curve according to different subgroups.

There was a curved association after adjusting for confounding factors in the Adjust Ⅱ model. We observed an inflection point in the saturation effect (Table 4). When NT-proBNP was < 1099 ng/L, it was associated with mortality (OR=1.10, 95%CI: 1.03-1.17, P=0.0025). When >1099 ng/L, there was no association (OR=1.00, 95%CI: 0.99-1.02, P=0.7786). Figure 2 shows the Kaplan-Meier survival curve. In the high NT-proBNP group, the one-year mortality rate was 14.87%.

**Table 4:** Nonlinearity of preoperative NT-proBNP versus mortality. Outcome variable: one-year mortality, Exposure variable: NT-proBNP, Adjusted for: age, sex, hypertension, CHD, cancer, hepatitis, urea, creatinine, transfusion, and stay in hospital. P<0.05 is considered significant.

Outcome	OR (95%CI)	P-value
Fitting model by stand linear regression	1.01 (1.00, 1.03)	0.0504
Fitting model by two-piecewise linear regression		
inflection point	1099 ng/L	
<1099 ng/L	1.10 (1.03, 1.17)	0.0025
>1099 ng/L	1.00 (0.99, 1.02)	0.7786
p for log-likelihood ratio test	0.009	

## Discussion

Serum NT-proBNP levels increase with age and may be related to changes in cardiac microstructure or diastolic function due to aging. The NT-proBNP levels in normal women are higher than in men. There are many conditions that the NT-proBNP will increase in geriatric hip fractures, such as advanced heart disease, lung diseases, anemia after the fracture, and liver and kidney function dysfunction.

The present study showed that patients with higher NT-proBNP concentrations had higher one-year mortality. Notably, there was a nonlinear association between NT-proBNP concentration and one-year mortality; a concentration of 1099 ng/L was an inflection point. When the NT-proBNP concentration was below 1099 ng/L, the mortality increased by 10% (OR=1.10, 95%CI: 1.03-1.17, P=0.0025) when NT-proBNP increased by 100 ng/L. When the NT-proBNP concentration was above 1099 ng/L, the mortality did not increase anymore when NT-proBNP increased (OR=1.00, 95%CI: 0.99-1.02, P=0.7786). Thus, NT-proBNP was a helpful indicator for predicting high one-year mortality in practice.

As we know, the level of NT-proBNP was close to increasing age, and the mortality rate was also related to age. In three years of follow-up, age was a crucial confounding factor in the previous study [18]. To thoroughly analyze the association between NT-proBNP and all-cause mortality, the short-term, primarily one-year mortality, was more important than long-term data. We found that the inflection point (1099 ng/L) in the short-term study was higher than in the long-term study [18]. In addition, we excluded the patients receiving non-surgical treatment to show the association in this study. Thus, we are more convinced about the association between NT-proBNP and all-cause mortality in geriatric hip fractures.

In the serum NT-proBNP and hip fracture, it was reported that NT-proBNP concentration could predict perioperative cardiac complications from an anesthetist's perspective [12]. Later, more authors began to focus on the role of NT-proBNP in hip fractures. Elevated NT-proBNP was reported to be associated with increased 30-day mortality [19] and all-cause mortality within 90 days [13]. These studies have built an association between NT-proBNP concentration and mortality. Some studies also gave cut-off values by manual grouping. It was reported that NT-proBNP >450 ng/L [20] or > 600 ng/L [21] was an independent preoperative risk factor for postoperative major adverse cardiovascular events. It was also reported that an elevated perioperative NT-proBNP >806 ng/L was an independent predictor of short- and long-term mortality than clinical risk scores [14]. In this study, we got the inflection point by a two-piecewise binary logistic regression model and found 1099 ng/L was a helpful indicator. This first study illustrates the natural curve association between NT-proBNP and one-year mortality. NT-proBNP, as a known predictor of poor outcomes in patients with acute coronary syndrome and myocardial ischemia, is one factor that may result in left ventricular systolic dysfunction and elevated NT-proBNP [22]. That could be one of the reasons why elevated NT-proBNP is closely correlated with the damage before the surgery and perioperative on the myocardium [23].

Additionally, we identified the factors affecting mortality to explore the possible confounders. The factors of age [3], male [2], hypertension [24], CHD [25], cancer [26], and length of stay in hospital [27] were identified in previous studies. Mainly, receiving a blood transfusion during admission for hip fracture carried an increased risk of one-year mortality of almost two and a half times [28]. We have found that hepatitis was associated with mortality in univariate analysis. Also, renal function may affect the NT-proBNP levels [29], and we included the urea and creatinine and adjusted these indicators to build the association between NT-proBNP and mortality.

In the future, we need more predictors and management to evaluate and reduce the incidence of complications and mortality. In clinical, a decrease in NT-proBNP correlates with a more favorable outcome in people with heart failure [30]. However, we did not find evidence of hip fracture or know the role of decreasing NT-proBNP on mortality. Thus, a controlled trial is needed to show the effect on mortality by reducing the concentration of NT-proBNP in the future.

Our study's one-year mortality rate was 9.1%, which is low compared with the world data [2-6]. In other studies [2-6], it is approximately 30%-35% for one year. The main reason may be that we take more time to adjust the patients' situation before the surgery. Thus, the time to operation is beyond 48 hours in our study.

The study had some advantages; firstly, we collected some patient's clinical data from the largest trauma center in northwestern China, extending this study's universality. Second, this study adjusted for a considerable number of confounding factors that can influence the association between NT-proBNP and one-year mortality after performing hip fracture surgery. Also, there are two limitations. Firstly, the loss to follow-up is 16.7%, even though we contacted the loss of patients more than three times. Second, NT-proBNP is a predictor of all-cause mortality, particularly cardiovascular deaths. We followed the patients by telephone and could not get the real cause of death. Thus, there is no data on cardiovascular deaths. Thirdly, the study's retrospective nature and the relatively small sample (in comparison with how many hip fractures are surgically treated annually) were other limitations.

## Conclusions

Because of the inflection point of 1099 ng/L, the NT-proBNP concentrations were nonlinearly related to one-year mortality in geriatrics hip fractures. Furthermore, NT-proBNP was a powerful predictor of subsequent all-cause mortality. NT-proBNP is a simple test before undergoing hip fracture operation that may be a valuable predictor to assess the preoperative risk and aid in clinical decisions on whether the surgery is performed and monitoring after the surgery. In addition, a well-designed controlled trial is needed to show the effect on mortality by decreasing the concentration of NT-proBNP in the future.
